# Endophytic Fungus* Aspergillus japonicus* Mediates Host Plant Growth under Normal and Heat Stress Conditions

**DOI:** 10.1155/2018/7696831

**Published:** 2018-12-06

**Authors:** Ismail, Muhammad Hamayun, Anwar Hussain, Amjad Iqbal, Sumera Afzal Khan, In-Jung Lee

**Affiliations:** ^1^Department of Botany, Abdul Wali Khan University Mardan 23200, Pakistan; ^2^Department of Agriculture, Abdul Wali Khan University Mardan 23200, Pakistan; ^3^Centre of Biotechnology and Microbiology, University of Peshawar, Pakistan; ^4^School of Applied Biosciences, Kyungpook National University, Daegu 41566, Republic of Korea

## Abstract

We have isolated an endophytic fungus with heat stress alleviation potential from wild plant* Euphorbia indica* L. The phylogenetic analysis and 18S rDNA sequence homology revealed that the designated isolate was* Aspergillus japonicus* EuR-26. Analysis of* A. japonicus* culture filtrate displayed higher concentrations of salicylic acid (SA), indoleacetic acid (IAA), flavonoids, and phenolics. Furthermore,* A. japonicus* association with soybean and sunflower had improved plant biomass and other growth features under high temperature stress (40°C) in comparison to endophyte-free plants. In fact, endophytic association mitigated heat stress by negotiating the activities of abscisic acid, catalase, and ascorbic acid oxidase in both soybean and sunflower. The nutritional quality (phenolic, flavonoids, soluble sugars, proteins, and lipids) of the* A. japonicus*-associated seedlings has also improved under heat stress in comparison to endophyte-free plants. From the results, it is concluded that* A. japonicus *can modulate host plants growth under heat stress and can be used as thermal stress alleviator in arid and semiarid regions of the globe (where mean summer temperature exceeds 40°C) to sustain agriculture.

## 1. Introduction

Temperature stress is becoming more persistent, especially in arid, semiarid, and tropical zones of the globe, causing great extortions to food crops. Rising in the total mean global temperature also results in saline conditions through moisture evaporation from the soil. Plants are vulnerable to various stresses because of their sessile nature. In such abnormal state, reactive oxygen species (ROS) are generated in high concentrations, which cause apoptosis or premature cell death when exposed for a longer time. Different ROS species, including singlet oxygen (^1^O_2_), superoxide (O_2_^−1^), hydroxyl radical (OH) and hydrogen peroxide (H_2_O_2_) containing free electrons, are leaked in chloroplast and mitochondria from electron transport chain [1]. ROS are trapped by biological membranes where they are detoxified by local antioxidants except H_2_O_2_ [2]. But all plants contain a specific enzymatic antioxidant system in the form of superoxide dismutase (SOD), catalase (CAT), ascorbic acid oxidase (AAO), and peroxidase (POD) which neutralize ROS before they come into action to damage the cell components [3]. CAT and AAO specifically target ROS by lowering their energy or disturbing their oxidizing chain reactions [4]. CAT is known to have multigene family responsible for encoding vital plant proteins. These vital proteins have significance in localization, regulation, and expression of stress receptive genes in cells facing different environmental stresses [5]. Similarly, AAO helps in controlling the level of membrane bound antioxidant *α*-tocopherol as well as reorganization of cell wall during different environmental stresses [6]. Moreover, plants also have the potential to modulate biochemical and physiological modifications depending on variable capacity to identify stimuli and conduct signals [7].

Phytohormones, like jasmonic acid (JA), abscisic acid (ABA), and salicylic acid (SA), serve as a signaling compound during stress [8]. Different phytohormones respond differently during biotic and abiotic stresses; for example, ABA regulates stomatal closure, plant growth, and development under stressful conditions [9]. SA controls plant growth, development, flower induction, stomatal response, ethylene synthesis, and respiration [10]. Similarly, JA is known to have a role in the biosynthesis of defensive secondary metabolites and proteins [11]. JA also helps in modulation of many physiological phenomena like resistance to insects and pathogen, pollen development, senescence, and root growth [12]. Endophytic fungi that reside inside plant tissues without causing any visible symptoms are one of the best bio agents that help in restoration of plant growth under stress [13]. They also have a role in enhancement of host growth, increasing of nutrient absorption, reduction in disease severity, and improving of host resistance against environmental stresses [13]. Vital secondary metabolites like hormones (GAs, IAA, SA, and JA), flavonoids, phenolics, and proline are also secreted by endophytic fungi in their culture filtrate and probably in their host tissues [14]. Plants lacking in endophytic fungi are more susceptible to drought, salinity, heat, and pathogenic stresses hence overall yield is highly reduced in such abnormal situations as compared to endophyte-hosted plants [15]. The present study was an attempt to explore such endophytic fungi that have the potential to improve host plant growth and yield as well as enhance their immunity under thermal stress.

## 2. Materials and Methods

### 2.1. Plant Sample Collection and Endophyte Isolation

A wild plant* Euphorbia indica* L. was collected from the desert area of District Noshera Khyber Pakhtunkhwa, Pakistan, and processed for the isolation of endophytic fungi using Khan et al. [16] procedure. Fungal isolates were purified on potato dextrose agar (PDA) medium and kept in refrigerator at 4°C. For longer storage, PDA slants were made [17]. For the collection and analysis of fungal secondary metabolites, endophyte isolates were grown in 50 ml of Czapek medium for 7 days in a shaking incubator set at 120 rpm at 28°C.

### 2.2. Screening Bioassay of fungal Filtrate on Rice Seedlings

Screening bioassay of fungal filtrate was carried out on rice seedlings by applying 100*μ*l of fungal filtrate on the tip of rice seedlings (Fakhr-e-Malakand variety provided by the Agricultural Biotechnology Institute, National Agricultural Research Center, Islamabad, Pakistan) at the two leaves stage grown in 0.8% water-agar medium in growth chamber (day/night cycle: 14 h, 28°C ± 0.3; 10 h, 25°C ± 0.3; relative humidity 70%) for 7 days. Root-shoot length and fresh and dry weights were analyzed after one week of filtrate treatment and compared with Czapek and distilled water treated plants.

### 2.3. Fungal Identification

For molecular identification of endophytic fungi, Khan et al. [16] method was applied. Internal transcribed region of 18S rRNA was amplified by using primers ITS1 (5′-TCC GTA GGT GAA CCT GCG G-3′) and ITS4 (5′-TCC TCC GCT TAT TGA TAT GC-3′). The sequence obtained, was then imperiled to BLASTn1 for sequence homology approximation. Neighbor joining (NJ) tree was applied for the phylogenetic analysis using the MEGA 7 software.

### 2.4. Inoculation of* Aspergillus japonicus *EuR-26 to Soybean and Sunflower

Endophytic fungus* A. japonicus* was grown in 250 ml conical flask containing 50 ml Czapek broth and kept in shaking incubator set at 120 rpm for 7 days at 28°C. Pellet and supernatant was separated using filter paper. One milligram of fungal biomass per 100 g of sterilized sand was inoculated to pots containing 9 seeds of soybean and sunflower each and shifted to growth chambers set at 25°C and 40°C for two weeks while supernatant was used for the determination of secondary metabolites. Half strength Hoagland solution (10 ml) was applied to the pots at 48 h intervals. Growth parameters were evaluated after 2 weeks of incubation in growth chamber [18]. The experiments were performed in triplicate (each replicate consisted of 9 seedlings).

### 2.5. IAA and SA Analysis in the Culture Filtrate of* A. japonicus*

For the determination of IAA in the filtrate of* A. japonicus* Benizri et al. [19] protocol was used. Two ml Salikowski reagent was mixed well with 1 ml of pure fungal filtrate and then kept in the dark for half an hour at 25°C. OD was taken at 540 nm with the help of PerkinElmer Lambda 25 spectrophotometer. Different concentrations (10, 20, 30, 40, 60, 80, and 100 *μ*g/ml) of the IAA (Sigma Aldrich) were used to construct the standard curve.

For the determination of SA the method of Warrier et al. [20] was followed. Exactly, 2.99 ml freshly prepared FeCl_3_ (0.1%) solution was mixed with 100 *μ*l of fungal filtrate. After the appearance of violet color, OD was observed at 540 nm. A standard curve was plotted by taking different concentrations (100, 200, 300, 400و and 500 *μ*g/ml) of pure SA (Sigma Aldrich).

### 2.6. ABA Analysis in Sunflower and Soybean

For the analysis of ABA contents in soybean and sunflower the protocol of Yoon et al. [21] was followed. Fresh leaves of soybean and sunflower (0.5 g each) were ground in liquid nitrogen. A mixture of isopropanol (1.5 ml) and glacial acetic acid (28.5 ml) was added to the grounded leaves. The mixture was filtered and dehydrated by means of a rotary evaporator. Diazomethane was then added to the mixture and analyzed by GC–MS SIM (6890N network GC system equipped with 5973 network mass selective detector; Agilent Technologies, Palo Alto, CA, USA). The Lab-Base (Thermo Quset, Manchester, UK) data system software was used to observe responses to ions with m/z values of 162 and 190 for Me-ABA and 166 and 194 for Me-[2H6]-ABA. ABA ([2H6]-ABA) from Sigma Aldrich was used as internal standard.

### 2.7. Analysis of Antioxidants in Soybean and Sunflower Seedlings

Luck [22] protocol was followed in the determination of CAT concentration in soybean and sunflower seedlings. Fresh leaves (2 g) of soybean and sunflower were crushed in phosphate buffer (10 ml) and centrifuged for five minutes at 10,000 rpm. H_2_O_2_-phosphate buffer (3 ml) was then added to the supernatant (40 *μ*l) and the OD was observed at 240 nm. H_2_O_2_-free phosphate buffer solution was used as blank. The enzyme unit was calculated as the amount of enzyme required to decrease the absorbance at 240 nm by 0.05. Oberbacher and Vines [23] protocol was used to measure the concentration of AAO in soybean and sunflower seedlings. Fresh leaves (0.1 g) of soybean and sunflower were ground in phosphate buffer (2 ml) and centrifuged for five minutes at 3000 rpm. About 3 ml of the substrate solution (8.8 mg of ascorbic acid in 300 ml phosphate buffer, pH 5.6) was mixed with 100 *μ*l of supernatant and the OD was noted after every 30 seconds till 5 minutes at 265 nm.

### 2.8. Analysis of Phenolics, Proline and Flavonoids in the Culture Filtrate of* A. japonicus*, Sunflower, and Soybean

Cai et al. [24] procedure was employed for the analysis of phenolics in soybean, sunflower, and fungal filtrate. Different concentrations (100, 200, 300, 500, 600, 700, and 900 mg/ml) of gallic acid (Sigma Aldrich) were used to construct a standard curve. For the determination of proline, the method of Bates et al. [25] was followed, but after some modifications. A standard curve was plotted against different concentrations (2, 4, 6, 8, and 10 *μ*g/ml) of standard proline (Sigma Aldrich) and the OD was taken at 520 nm. For the analysis of total flavonoids, the method of Mervat et al. [26] was used. A standard curve was constructed by using different concentrations of pure quercetin (15, 30, 60, 120, 240, and 480 *μ*g/ml, Sigma Aldrich) and the OD were taken at 415 nm.

### 2.9. Analysis of Total Proteins, Lipids, and Soluble Sugars

Lowry et al. [27] method was employed for the analysis of total proteins in the seedlings of soybean and sunflower. A standard curve was drawn using different concentrations (20, 40, 60, 80, and 100 *μ*g/ml) of BSA (Sigma Aldrich) and the OD was observed at 650 nm. For the determination of total lipids, we used the method of Van Hand et al. [28] with some modifications. A standard curve was constructed using different concentrations of canola oil (10, 40, 70, 100, 130, and 160 *μ*g/ml) and the OD was noticed at 490 nm. For the analysis of total soluble sugars in the soybean and sunflower seedlings, the method of Mohammadkhani and Heidari [29] was used. A standard curve was constructed by using different concentrations (20, 40, 60, 80, and 100 *μ*g/ml) of glucose (Sigma Aldrich) and the OD was measured at 485 nm.

### 2.10. Statistical Analysis

All the experiments were performed in triplicate. ANOVA (one-way analysis of variance) was used for the analysis of data and means were compared by Tukey HSD test at p < 0.05, using SPSS-20 (SPSS Inch., Chicago, IL, USA) for windows.

## 3. Results

### 3.1. Endophytes Isolation and Their Preliminary Screening on Rice Seedlings

We isolated 14 different endophytic fungi (Figure S1) from the wild plant of* Euphorbia indica *L. and initially screened on rice seedlings for their growth promoting or inhibiting activities (Figure S2). Endophytes were first isolated on Hagem minimal medium and then purified on PDA on the basis of morphological differences. After screening on rice seedlings at the two leaves stage, growth parameters were recorded after 1 week of filtrate treatment (Table 1). EuR-26 isolate was found to be growth promoter and chosen for molecular identification.

### 3.2. Molecular Identification of Fungal Isolate EuR-26

**Figure 1:**
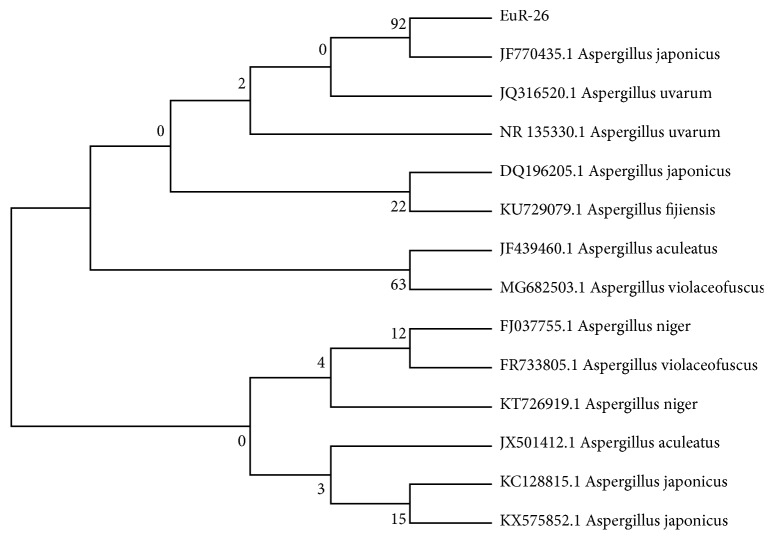
Phylogenetic consensus tree using neighbor joining (NJ) method for the identification of isolate EuR-26, using 14 taxa, 13 references, and 1 clone. The isolate was identified as* Aspergillus japonicus* as having 92% bootstrap value.

Fresh mycelium was used for the extraction of genomic DNA while, applying ITS1 (5′-TCC GTA GGT GAA CCT GCG G-3′) and ITS4 (5′-TCC TCC GCT TAT TGA TAT GC-3′). Sequence of ITS region of related fungi was compared with the nucleotide sequence of our fungal isolate EuR-26 with the help of the BLAST search program. Sequence of 18S rDNA exhibited maximum similarity (92%) with* A. Japonicas*. The phylogenetic consensus tree was built from 14 (13 reference and 1 clone) by neighbor joining (NJ) method using MEGA 7 software (Figure 1). Our isolate EuR-26 made a clad with* A. japonicus* supported by 92% bootstrap value in the consensus tree. Combining results of phylogenetic analysis and sequence homology suggested that the strain EuR-26 was* A. japonicus*.

### 3.3. Analysis of Secondary Metabolites in the Culture Filtrates of* A. japonicus*

**Figure 2:**
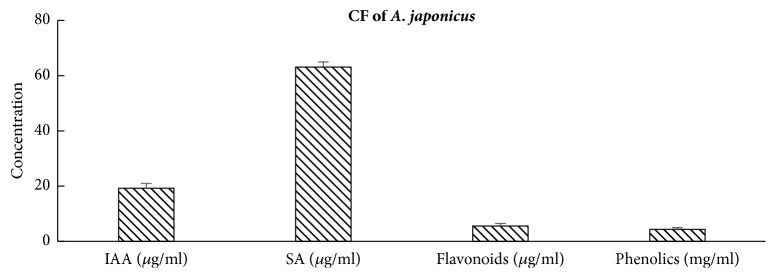
Analysis of secondary metabolites secreted by* A. japonicus* (EuR-26) in their culture filtrate (CF) grown for 7 days in Czapek medium in shaking incubator set at 120 rpm at 28°C. The bars labeled with different letters are significantly different at p < 0.05 as estimated by Tukey HSD test. The bars represent SE of a triplicate data (each replicate consisted of 9 seedlings).

Culture filtrate of* A. japonicus *was analyzed for the presence of important secondary metabolites including IAA, SA, flavonoids, and phenolics. Different concentrations of secondary metabolites were IAA (19.19 *μ*g/ml), SA (63.11*μ*g/ml), flavonoids (5.56 *μ*g/ml), and phenolic (4.4 mg/ml) (Figure 2).

### 3.4. Growth Features of* A. japonicus*-Associated Soybean and Sunflower

Both* A. japonicus*-associated soybean and sunflower at 25°C and 40°C showed significant increase in various growth parameters, including chlorophyll content, shoot length, shoot fresh, and dry weights as compared to endophyte-free plants. At room temperature (25°C) soybean (Table 2) and sunflower (Table 3) seedlings have more chlorophyll, root-shoot length, and dry weight than under heat stress condition (40°C).

### 3.5. Modulation in* A. japonicus*-Associated Soybean and Sunflower's Endogenous Flavonoids, Phenols, and Proline

**Figure 3:**
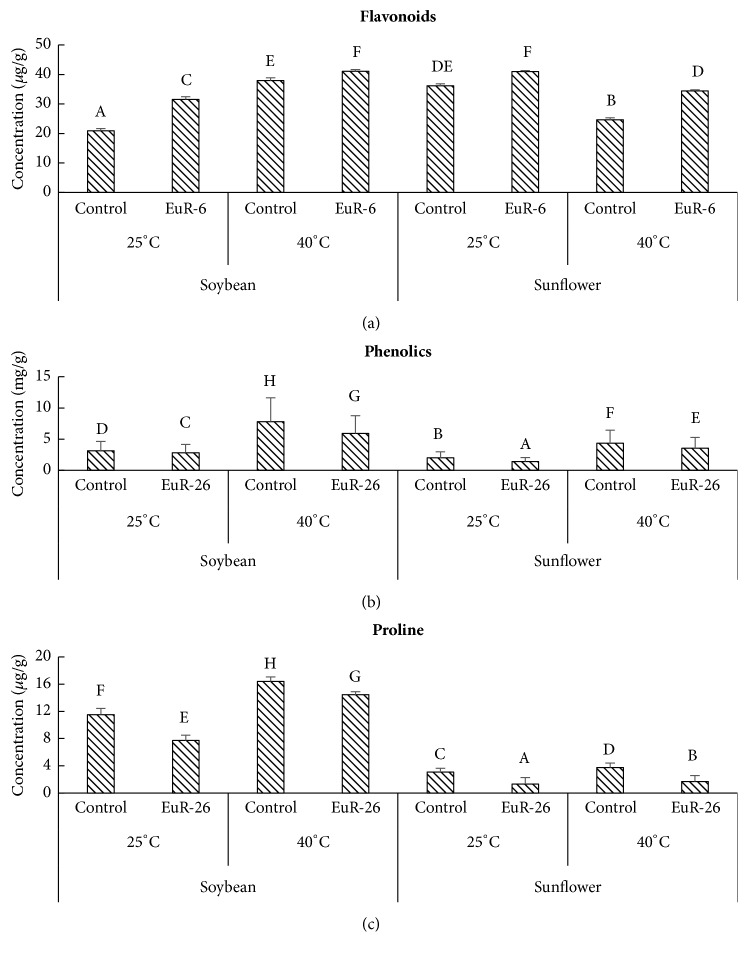
Effect of* A. japonicus* on the concentration of (a) flavonoid, (b) phenolics, and (c) proline in soybean and sunflower seedlings grown at 25°C and 40°C. For each set of treatment, the different letter indicates significant differences at p (< 0.05) as estimated by Tukey HSD test. The bars represent SE of a triplicate data (each replicate consisted of 9 seedlings).

Important secondary metabolites like flavonoids, phenolics, and prolines were analyzed in soybean and sunflower seedlings inoculated with* A. japonicus *at 25°C and 40°C in growth chamber. An increase has been noted in* A. japonicus-*associated (41.13 *μ*g/g) and control (37.96 *μ*g/g) soybean seedlings at 40°C as compared at 25°C (31.53 *μ*g/g in experimental and 20.91*μ*g/g in control seedlings), while endophyte-aligned seedlings have more flavonoids content as compared to the endophyte-free seedlings. Similarly, sunflower seedlings aligned with* A. japonicus* have more flavonoids (41.04 *μ*g/g at 25°C and 34.43 *μ*g/g at 40°C) than control plants (36.18 *μ*g/g at 25°C and 24.64 *μ*g/g at 40°C) at both temperatures, while a decrease has been noted at thermal stress as compared to normal condition (Figure 3(a)). We also found a slight reduction in the phenolics and proline concentration in* A. japonicus*-aligned soybean and sunflower seedlings as compared to control plants. At 25°C endophyte-aligned soybean has 2.8 mg/g and endophyte-free seedlings have 3.13 mg/g of phenolics while, at 40°C, endophyte-aligned soybean has 5.89 mg/g and endophyte-free seedlings have 7.8 mg/g of phenolics. Sunflower seedlings inoculated with* A. japonicus* at 25°C have 1.38 mg/g and control plants have 2.01 mg/g of total phenolics while, at 40°C, endophyte-associated sunflower has 3.53 mg/g and endophyte-free seedlings have 4.34 mg/g of phenolics (Figure 3(b)). A significant reduction was found in the total proline concentration in soybean (7.71 *μ*g/g at 25°C and 14.46 *μ*g/g at 40°C) and sunflower (1.29 *μ*g/g at 25°C and 1.68 *μ*g/g at 40°C) seedlings aligned with* A. japonicus* as compared to control soybean (11.5 *μ*g/g at 25°C and 16.4 *μ*g/g at 40°C) and sunflower (3.09 *μ*g/g at 25°C and 3.73 *μ*g/g at 40°C) seedlings (Figure 3(c)).

### 3.6. Reduction in the Concentration of CAT and AAO

**Figure 4:**
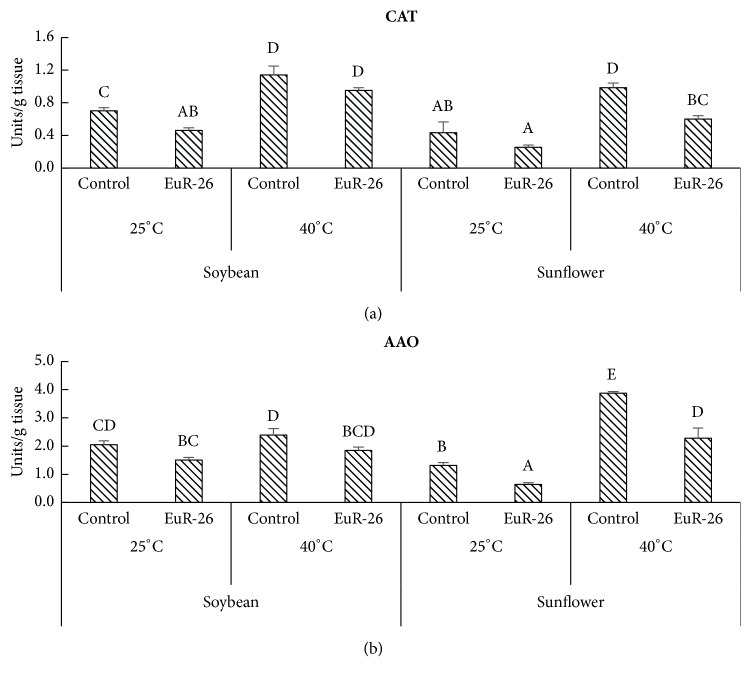
Effect of* A. japonicus* on the concentration of (a) CAT and (b) AAO in soybean and sunflower seedlings grown at 25°C and 40°C. One enzyme unit was calculated as the amount of enzyme required to decrease the absorbance at 240 nm for CAT and 265 nm for AAO by 0.05 units. For each set of treatment, the different letter indicates significant differences at p (< 0.05) as estimated by Tukey HSD test. The bars represent SE of triplicate data (each replicate consisted of 9 seedlings).

A significant reduction was detected in the concentration of CAT and AAO in soybean and sunflower seedlings associated with* A. japonicus* as compared to control plants. Endophyte-aligned soybean has 0.46 enzyme unit/g tissue and sunflower had 0.25 enzyme unit/g tissue CAT at 25°C as compared to endophyte-free soybean 0.7 enzyme unit/g tissue and sunflower 0.43 enzyme unit/g tissue. At 40°C, the soybean seedlings had 0.95 enzyme unit/g tissue and sunflower had 0.6 units of enzyme unit/g tissue as compared to control soybean 1.14 enzyme unit/g tissue and sunflower 0.98 enzyme unit/g tissue (Figure 4(a)). A similar decrease was found in the amount of AAO in soybean and sunflower aligned with* A. japonicus *at 25°C and 40°C as compared to endophyte-free soybean and sunflower (Figure 4(b)).

### 3.7. Reduction in Endogenous ABA Content in Soybean and Sunflower

**Figure 5:**
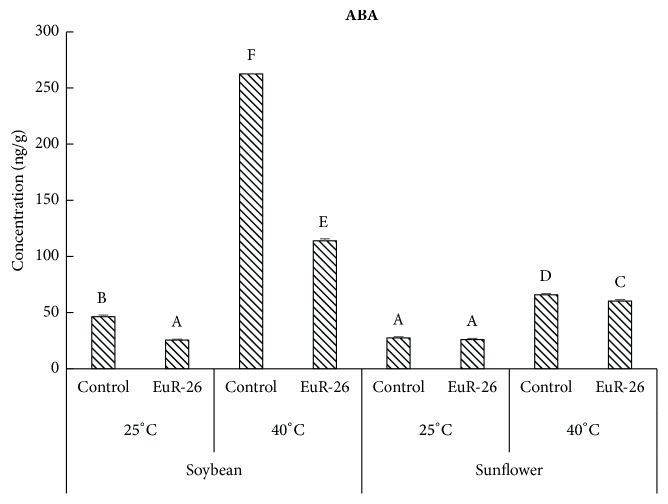
GC MS quantification of endogenous ABA concentration in soybean and sunflower grown at 25°C and 40°C with and without* A. japonicus*. For each set of treatment, the different letter indicates significant differences at p (< 0.05) as estimated by Tukey HSD test. The bars represent SE of a triplicate data (each replicate consisted of 9 seedlings).

A significant reduction was found in the concentration of ABA in soybean and sunflower seedlings associated with* A. japonicus* as compared to control seedlings at 40°C. Endophyte-associated soybean has 25.56 ng/g and endophyte-free seedling had 46.42 ng/g at 25°C. At 40°C, the experimental soybean had 113.78 ng/g and control seedling has 262.46 ng/g of ABA concentrations. Similarly, Endophyte-associated sunflower has 26.05 ng/g and endophyte-free seedling has 27.31 ng/g at 25°C while, at 40°C, experimental sunflower has 60.32 ng/g and control seedling has 65.78 ng/g of ABA concentrations (Figure 5).

### 3.8. Enhancement in the Nutritive Value of Soybean and Sunflower

**Figure 6:**
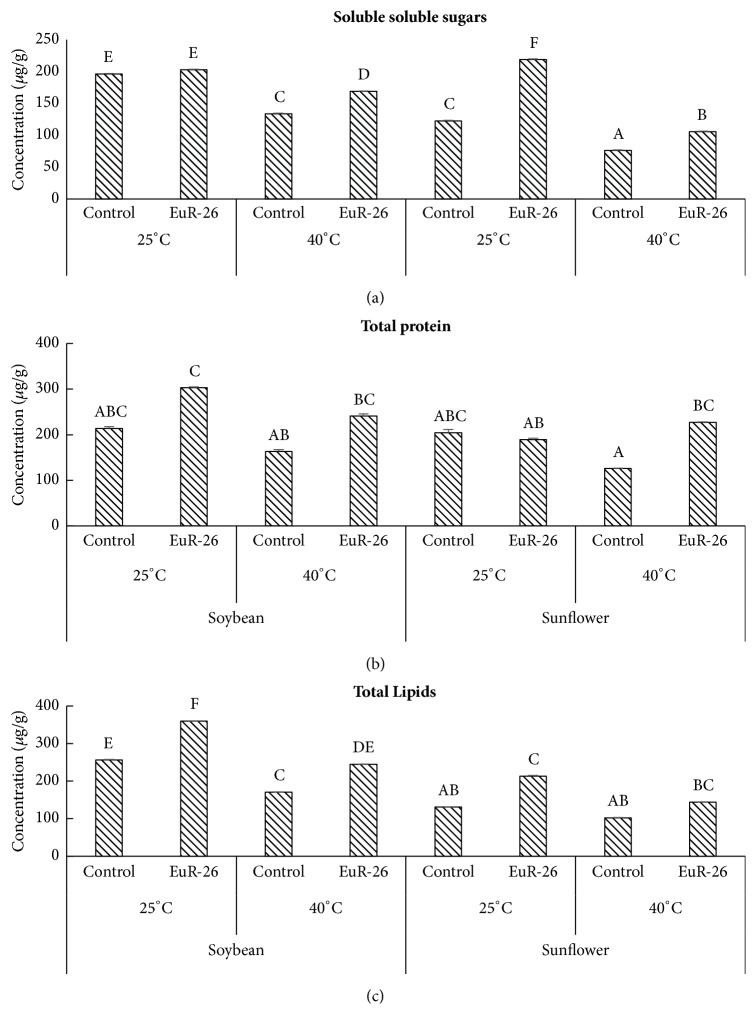
Analysis of (a) soluble sugars, (b) proteins, and (c) lipids of soybean and sunflower seedlings grown at 25°C and 40°C with- and without-endophytic fungal strain* A. japonicus*. For each set of treatment, the different letter indicates significant differences at p (< 0.05) as estimated by Tukey HSD test. The bars represent SE of a triplicate data (each replicate consisted of 9 seedlings).

Nutritive values of soybean and sunflower were determined by quantifying total sugar, protein, and lipid contents.* A. japonicus-*associated soybean has 202.8 *μ*g/g while control plants have 196.39 *μ*g/g of soluble sugars at 25°C and, at 40°C, experimental soybean seedlings had 169.03 *μ*g/g while control seedlings have 133.75 *μ*g/g total sugar content. A similar increase was observed in the total sugar concentrations of sunflower seedlings inoculated with an endophytic fungus as compared to endophyte-free seedlings under heat stress (Figure 6(a)). A significant increase was detected in the total protein contents of soybean (302.83 *μ*g/g at 25°C and at 240.8 *μ*g/g 40°C) and sunflower (189.15 *μ*g/g at 25°C and at 227.29 *μ*g/g 40°C) aligned with* A. japonicus *as compared control seedlings of soybean (213.67 *μ*g/g at 25°C and at 163.04 *μ*g/g 40°C) and sunflower (204.13 *μ*g/g at 25°C and at 125.84 *μ*g/g 40°C) (Figure 6(b)). An enhancement in the total lipids was found in* A. japonicus*-inoculated soybean and sunflower as compared to* A. japonicus-*free soybean and sunflower at 25°C and 40°C (Figure 6(c)).

## 4. Discussion

Endophytic fungi are known to have symbiotic association with plants in natural ecosystem [30]. The endophytes are responsible for synthesizing a large number of vital bioactive secondary metabolites, including phenolics, flavonoids, and alkaloids, that have importance in the fields of medicine, food, and agriculture [31]. Endophytes have a defensive role for their host plant in providing resistance against abiotic and biotic stresses as well as improving overall growth and yield [32]. We isolated endophytic fungi from wild plant* Euphorbia indica* L. found in xeric condition and screened them for their growth promoting potential under thermal stress. Fungal filtrate was initially screened on rice seedlings (Fakhr-e-Malakand variety provided by the Agricultural Biotechnology Institute, National Agricultural Research Center, Islamabad, Pakistan) for their growth promoting or inhibiting activities. The rice was selected because their response is very easy and quick to growth hormones, like GAs and IAA, secreted by endophytic fungi [33]. Culture filtrate of* A. japonicus* significantly enhanced rice growth features and suggested that this endophyte has the potential for plant growth promotion, which was later on confirmed by detecting IAA in their culture filtrate. Plant growth promoting potential of endophytic fungi and bacteria was previously reported by Mei and Flinn [34]. Endophytic fungi improve plant growth and development by secreting bioactive secondary metabolites and sharing of mutualistic genes that can work better for host performance [32]. Prolonged exposure of plants to different stresses like drought, salinity, and heat results in growth inhibition, acceleration of apoptosis, and premature cell death [35]. Present study on the association of* A. japonicus *with roots of soybean and sunflower caused a significant increase in chlorophyll content, root-shoot length, and biomass of host plants. The increase in chlorophyll contents in endophyte-associated plants might be associated with increased photosynthetic rate. Sun et al. [36] reported similar observations that the endophyte* Piriformospora indica* successfully inhibited the drought initiated losses in chlorophyll contents by maintaining the photosynthetic rate. Similarly, plant endophytes have been noticed to promote plant growth and biomass under abiotic stress conditions.* Penicillium resedanum, Curvularia protuberate, Alternaria *sp.*, and Trichoderma harzianum* are one of the best examples that have controlled the shoot and root lengths and biomass production of the host plants under abiotic stress [37–39]. The host plant growth promotion by the endophytes under abiotic stress can be attributed to the IAA and GA production [40–42]. This means that* A. japonicus *as an endophyte helped the host plants (soybean and sunflower) to grow normal under heat stress, i.e., 40°C. Also, it is possible that the IAA producing* A. japonicus* might also be helpful in alleviating biotic and abiotic stresses in host plant species. Our results strongly supported and confirmed the findings of Mei and Flinn [34], who reported that IAA producing fungal and bacterial endophytes can improve rice growth under drought, salinity, and high temperature stress. Besides the plant growth promotion,* A. japonicas* have also improved the nutritional quality of both soybean and sunflower under stress condition. The* A. japonicas* associated soybean and sunflower seedlings have higher amounts of soluble sugars, total proteins, and total lipids as compared to the nonassociated seedlings of both species, when grown under 40°C. Similar observations have been noted by Clifton et al. [43], who said that the alleviation in nutritional quality of soybean was due to fungi,* M. brunneum*. Certainly, the endophytes might help the host plants to absorb optimum amounts of nutrients from rhizosphere to fulfill its nutrient requirements even under stress conditions.

Signaling by phytohormones especially ABA is one of the important defense strategies by higher plants under abiotic stresses. ABA significantly increases during abiotic stresses, like high temperature in rice seedlings [44]. Heat stress cause upregulation of genes responsible for ABA biosynthesis, while it downregulates the genes involved in ABA catabolism [45]. We found low levels of ABA in* A. japonicus*-aligned soybean and sunflower exposed to thermal stress as compared to control plants, indicating that endophytic fungi have the capacity to lower the effect of heat stress for host plants.

Low concentration of ROS is required for better signaling, growth and development while their high concentration is injurious to plant tissues leading to premature cell death. Plants display a remarkable collection of enzymatic and nonenzymatic antioxidant defense systems that neutralize ROS toxicity in stressful conditions [46]. CAT is known to be involved directly while AAO as indirectly in ROS detoxification. High concentration of antioxidants, including CAT and AAO, determines stress severity and susceptibility in plants [47]. A significant decrease was found in the activities of CAT and AAO in* A. japonicus*-associated soybean and sunflower under thermal stress as compared to endophyte-free plants. CAT and AAO have a role in degrading excess of H_2_O_2_ formed in microbodies and mitochondria of plants under stress as well as regulate stress responses in plants. Our results confirmed the work of Waqas et al. [10], who observed that endophytes-hosted plants are more resistant to environmental stresses than endophyte-free plants.

Plants accumulate high content of proline in response to different environmental stresses as an osmolyte, a buffering agent for cellular redox and a scavenger of free radicals potential [48]. It also plays a role in alleviating cytoplasmic acidosis and keeping proper proportion of NADP+/NADPH necessary for harmonious plant-cellular-metabolism [49] and proteins [50]. After the harsh stressful condition, hoarded proline quickly disintegrates, releasing high concentration of powerful reducing agents, which speed up ATP synthesis and renovation of stress-induced damage [51]. Some abiotic stress responsive genes that have proline responsive elements, like PRE, ACTCAT in their promoter regions, are also expressed by proline accumulated under stress [52]. High concentration of proline in our controlled soybean and sunflower as compared to* A. japonicus*-associated plants confirmed that proline accumulation enhances under abiotic stresses including high temperature [53], while endophytic fungi help in reducing the stress severity for the host plant.

Phenolics are natural compounds synthesized in response of changing environmental conditions and essential for plants in defensive mechanisms [54]. Different phenolic compounds are accumulated by higher plants as defense tools against different biotic and abiotic stresses [55]. Accumulation of phenolics by plants during stressful condition is one of the common indicators of plants. Thus, our results that temperature stress enhances phenolics in soybean and sunflower strongly support the research work of Lattanzio et al. [56]. Moreover, decrease in the amount of phenolics in* A. japonicus*-associated soybean and sunflower suggests that endophytic fungi have a role in alleviating high temperature stress in host plants. On the contrary, the amounts of flavonoids increased in* A. japonicus*-associated soybean and sunflower seedlings in comparison to the* A. japonicus-*free seedlings. From the results, we can argue that* A. japonicas* can ameliorate the production of flavonoids in both species to improve its health protective effect on human beings. The results of the present study are in accordance with the observations of Yang et al. [57], who observed high total flavonoids in fungal inoculated wine grapes.

## 5. Conclusion

The current study revealed that endophyte-associated plants have promoted the growth of soybean and sunflower seedlings under normal as well as high temperature stress as compared to endophyte-free plants. Moreover, the interaction of endophytic fungi with economically vital crops can significantly improve their nutritive quality and quantity. Hence, the uses of endophytic fungi not only promote growth of the plants under normal condition, but also alleviate thermal stress in crop plants. Therefore, the application of* A. japonicus* to crop plants is suggested in the future for sustainable agriculture.

## Figures and Tables

**Table 1:** Effect of *A. japonicus *filtrate on the growth of rice seedlings.

**Growth attributes**	**Control (DW)**	**Control (Czk)**	***A. japonicus***
Shoot Length (cm)	12.6 ± 0.25 ^a^	13.8 ± 0.34 ^ab^	15.67 ± 0.52 ^b^
Root Length (cm)	6.3 ± 0.38 ^a^	6.6 ± 0.18 ^ab^	8.1 ± 0.35 ^b^
Dry Weight Shoot (g)	0.0034 ± 0.001 ^a^	0.0049 ± 0.003 ^a^	0.0086 ± 0.004 ^b^
Dry Weight Root (g)	0.0147 ± 0.001 ^a^	0.0155 ± 0.004 ^a^	0.0182 ± 0.004 ^b^
Chlorophyll (SPAD)	20.73 ± 1.5 ^a^	22.7 ± 0.56 ^b^	25.9 ± 0.18 ^c^

DW = distilled water, Czk = Czapek broth medium. Data are means of 3 replicates (each replicate consisted of 9 seedlings) with standard error. For each set of treatment, the different letter indicates significant differences at p (< 0.05) as estimated by Tukey HSD test.

**Table 2:** Effect of *A. japonicus* on the growth features of soybean.

**Growth attributes **	**25**°**C**		**40**°**C**	
	**Control**	***A. japonicus***	**Control**	***A. japonicus***
Chlorophyll (SPAD)	32.6 ± 0.21 ^a^	37.4 ± 0.4 ^d^	29 ± 2.46 ^a^	33.2 ± 0.07 ^c^
Shoot Length (cm)	38.7 ± 1.76 ^b^	48.3 ± 1.2 ^e^	26 ± 0.93 ^a^	35.5 ± 1.26 ^bc^
Root Length (cm)	16.3 ± 3.9 ^abc^	13.3 ± 2.33 ^ab^	10 ± 0.6 ^ab^	12 ± 2.6 ^abcd^
Dry Weight Shoot (g)	0.14 ± 0.01 ^a^	0.147 ± 0.01 ^ab^	0.08 ± 0.0003 ^ab^	0.09 ± 0.0001 ^ab^
Dry Weight Root (g)	0.08 ± 0.002 ^ab^	0.10 ± 0.01 ^bc^	0.01 ± 0.0001 ^ab^	0.062 ± 0.001 ^e^

Data are means of three replicates (each replicate consisted of 9 seedlings) with standard errors. For each set of treatment, the different letter indicates significant differences at p (< 0.05) as estimated by Tukey HSD test.

**Table 3:** Effect of *A. Japonicas* on the growth features of sunflower.

**Growth attributes **	**25**°**C**		**40**°**C**	
	**Control**	***A. japonicus***	**Control**	***A. japonicus***
Chlorophyll (SPAD)	39 ± 1.29 ^a^	43.3 ± 0.6 ^ab^	38.4 ± 1.95 ^a^	39.9 ± 3.12 ^a^
Shoot Length (cm)	23.7 ± 0.88 ^a^	25.2 ± 0.43 ^ab^	20.5 ± 0.45 ^a^	21 ± 1.53 ^ab^
Root Length (cm)	9 ± 0.58 ^a^	11.6 ± 0.87 ^abcd^	6 ± 0.58 ^b^	6 ± 0.25 ^b^
Dry Weight Shoot (g)	0.085 ± 0.009 ^ab^	0.088 ± 0.007 ^ab^	0.04 ± 0.0006 ^b^	0.037 ± 0.0002 ^a^
Dry Weight Root (g)	0.024 ± 0.001 ^a^	0.028 ± 0.002 ^a^	0.014 ± 0.0003 ^b^	0.02 ± 0.0002 ^c^

Data are means of three replicates (each replicate consisted of 9 seedlings) with standard errors. For each set of treatment, the different letter indicates significant differences at p (< 0.05) as estimated by Tukey HSD test.

## Data Availability

The authors confirm that the whole data is presented in the manuscript and supplementary files.

## Supplementary Materials

Supplementary Materials Supplementary 1. Figure S1: colonies of endophytic fungi grown on Hagem minimal medium and purified on PDA media plates, isolated from* Euphorbia indica* L. Different fungal colonies (14) were isolated from the host plant, 9 from roots, and 5 from stem; i.e., EuR-1, EuR-2, EuR-3, EuR-4, EuR-5, EuR-6, EuR-8, EuR-23, EuR-26, EuS-1, EuS-2, EuS-3, EuS-5, and EuS-14 (EuR represents* Euphorbia indica* L. root while EuS represents* Euphorbia indica* L. stem). Supplementary 2. Figure S2: screening bioassay of fungal culture filtrates (100*μ*l) isolated from* Euphorbia indica* L. on rice seedlings at 2 leaves stage grown in 0.8% water-agar medium for 2 weeks at 25°C. Reading taken after 1 week of culture filtrate application. 14 sets of pots (3 pots in each set) are shown. Each set has 3 treatments including Czapek control (right), distilled water control (left), and endophyte cultural filtrate (middle).
