# Supplementary material for: Endophytic Fungus Aspergillus japonicus Mediates Host Plant Growth under Normal and Heat Stress Conditions
Source: Biomed Res Int. 2018 Dec 6;2018:7696831. doi: 10.1155/2018/7696831 (PMC6304497; doi:10.1155/2018/7696831)
Supplement: Supplementary Materials — Supplementary 1. Figure S1: colonies of endophytic fungi grown on Hagem minimal medium and purified on PDA media plates, isolated from Euphorbia indica L. Different fungal colonies (14) were isolated from the host plant, 9 from roots, and 5 from stem; i.e., EuR-1, EuR-2, EuR-3, EuR-4, EuR-5, EuR-6, EuR-8, EuR-23, EuR-26, EuS-1, EuS-2, EuS-3, EuS-5, and EuS-14 (EuR represents Euphorbia indica L. root while EuS represents Euphorbia indica L. stem). Supplementary 2. Figure S2: screening bioassay of fungal culture filtrates (100μl) isolated from Euphorbia indica L. on rice seedlings at 2 leaves stage grown in 0.8% water-agar medium for 2 weeks at 25°C. Reading taken after 1 week of culture filtrate application. 14 sets of pots (3 pots in each set) are shown. Each set has 3 treatments including Czapek control (right), distilled water control (left), and endophyte cultural filtrate (middle). [file 7696831.f1.docx]

**
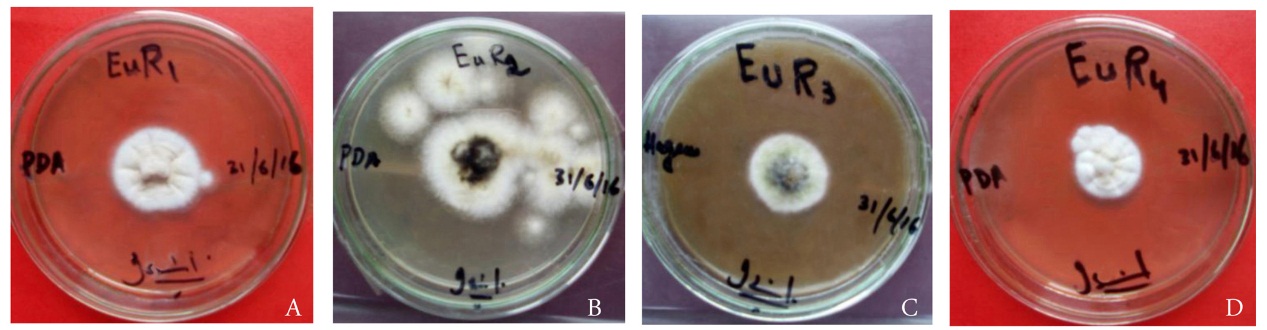
**

**
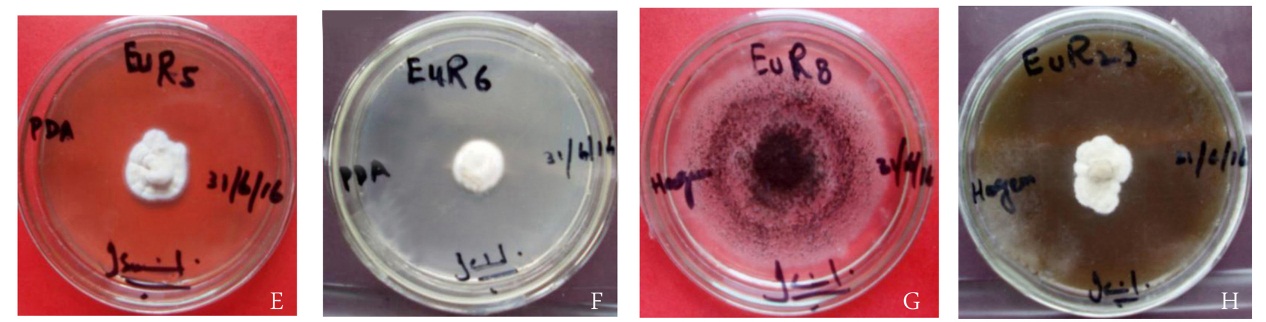
**

**
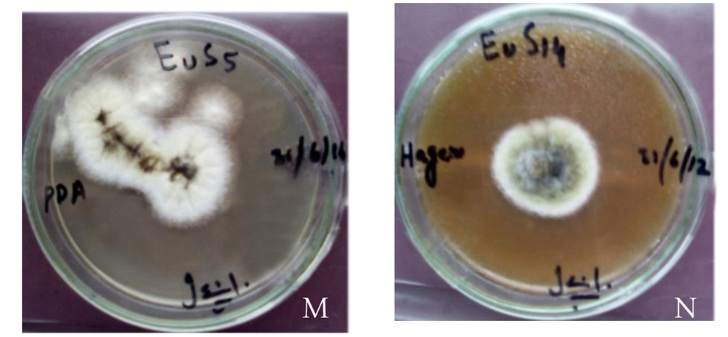

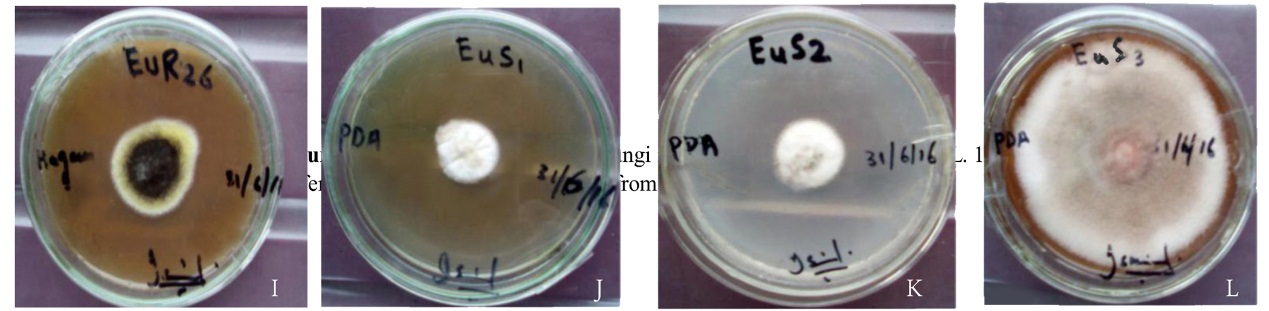
**

**Figure S1:** Colonies of endophytic fungi grown on Hagem minimal medium and purified on PDA media plates, isolated from *Euphorbia indica* L. Different fungal colonies (14) were isolated from the host plant, 9 from roots and 5 from stem i.e. EuR-1, EuR-2, EuR-3, EuR-4, EuR-5, EuR-6, EuR-8, EuR-23, EuR-26, EuS-1, EuS-2, EuS-3, EuS-5 and EuS-14 (EuR represents *Euphorbia indica* L. root while, EuS represents *Euphorbia indica* L. stem).

**
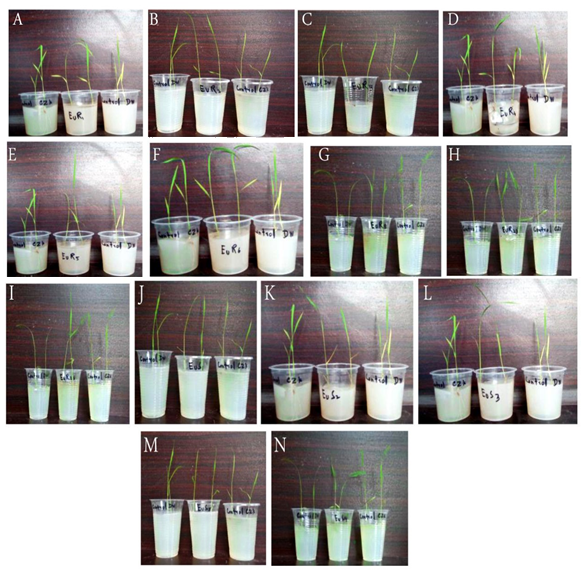
**

**Figure S2:** Screening bioassay of fungal culture filtrates (100μl) isolated from *Euphorbia indica* L. on rice seedlings at 2 leaves stage grown in 0.8% water-agar medium for 2 weeks at 25^o^C. Reading taken after 1 week of culture filtrate application. 14 sets of pots (3 pots in each set) are shown. Each set has 3 treatments including Czapek control (right), distilled water control (left) and endophyte cultural filtrate (middle).
